# Supplementary material for: The interactome of intact mitochondria by cross-linking mass spectrometry provides evidence for coexisting respiratory supercomplexes
Source: Mol Cell Proteomics. 2017 Dec 8;17(2):216–32. doi: 10.1074/mcp.RA117.000470 (PMC5795388; doi:10.1074/mcp.RA117.000470)
Supplement: Supplemental Data [file supp_RA117.000470_133922_0_supp_23978_fzffwf.zip › spectra_annotation/mito_spectra_annotation/145-1-4-1-8-1.pdf]

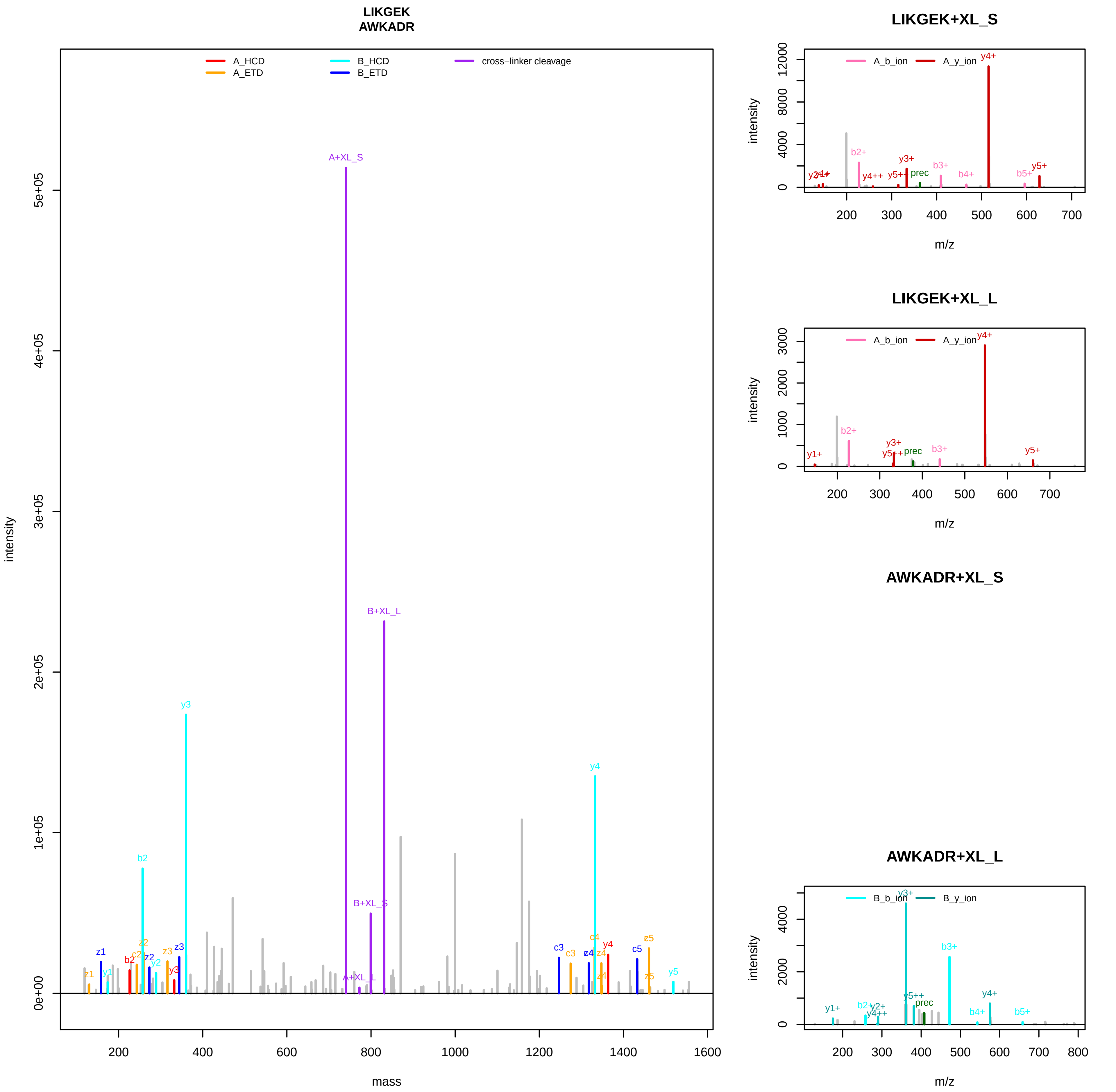
LIKGEK
AWKADR
A_HCD
A_ETD
B_HCD
B_ETD
cross−linker cleavage
A+XL_S
B+XL_L
B+XL_S
A+XL_L
y3
y4
b2
z1 y1 b2 c2 z2 z2 y2 z3 c3 y3 z3
c3 c3 c4 c4 z4 z4 y4 c5 z5 z5 y5
intensity
mass
0e+00
1e+05
2e+05
3e+05
4e+05
5e+05
200 400 600 800 1000 1200 1400 1600
LIKGEK+XL_S
A_b_ion
A_y_ion
y4+ y3+ b2+ b3+ b4+ b5+ y5+ y4++ y5++ prec
0 4000 8000 12000
200 300 400 500 600 700
m/z
LIKGEK+XL_L
A_b_ion
A_y_ion
y4+ b2+ y3+ y5++ prec b3+ y5+ y1+
0 1000 2000 3000
200 300 400 500 600 700
m/z
AWKADR+XL_S
AWKADR+XL_L
B_b_ion
B_y_ion
y3+ b3+ y5++ prec y4+ y1+ b2+ y2+ y4++ b4+ b5+
0 2000 4000
200 300 400 500 600 700 800
m/z
